# Gastric Fluid Metabolomics Predicting the Need for Surfactant Replacement Therapy in Very Preterm Infants Results of a Case–Control Study

**DOI:** 10.3390/metabo14040196

**Published:** 2024-03-30

**Authors:** Konstantia Besiri, Olga Begou, Konstantinos Lallas, Angeliki Kontou, Eleni Agakidou, Olga Deda, Helen Gika, Eleni Verykouki, Kosmas Sarafidis

**Affiliations:** 1 1st Department of Neonatology, School of Medicine, Aristotle University of Thessaloniki, Hippokration General Hospital, 54642 Thessaloniki, Greece; kmpesiri@auth.gr (K.B.); angiekon2001@yahoo.gr (A.K.); eagakidou@auth.gr (E.A.); 2 School of Chemistry, Aristotle University of Thessaloniki, 54124 Thessaloniki, Greece; mpegolga@chem.auth.gr; 3 Biomic_AUTh, Center for Interdisciplinary Research and Innovation (CIRI-AUTH), 57001 Thessaloniki, Greece; olgadeda@auth.gr (O.D.); gkikae@auth.gr (H.G.); 4 Department of Medical Oncology, School of Medicine, Aristotle University of Thessaloniki, Papageorgiou General Hospital, 56429 Thessaloniki, Greece; kplallas@auth.gr; 5 Laboratory of Forensic Medicine and Toxicology, School of Medicine, Aristotle University of Thessaloniki, 54124 Thessaloniki, Greece; 6 Laboratory of Biometry, University of Thessaly, 38446 Volos, Greece; everykouki@uth.gr

**Keywords:** gastric fluid, RDS, GC-MS, prematurity, surfactant, prediction

## Abstract

Respiratory distress syndrome (RDS) is a major morbidity of prematurity. In this case–control study, we prospectively evaluated whether untargeted metabolomic analysis (gas chromatography–mass spectrometry) of the gastric fluid could predict the need for surfactant in very preterm neonates. 43 infants with RDS necessitating surfactant (cases) were compared with 30 infants who were not treated with surfactant (controls). Perinatal–neonatal characteristics were recorded. Significant differences in gastric fluid metabolites (L-proline, L-glycine, L-threonine, acetyl-L-serine) were observed between groups, but none could solely predict surfactant administration with high accuracy. Univariate analysis revealed significant predictors of surfactant administration involving gastric fluid metabolites (L-glycine, acetyl-L-serine) and clinical parameters (gestational age, Apgar scores, intubation in the delivery room). Multivariable models were constructed for significant clinical variables as well as for the combination of clinical variables and gastric fluid metabolites. The AUC value of the first model was 0.69 (95% CI 0.57–0.81) and of the second, 0.76 (95% CI 0.64–0.86), in which acetyl-L-serine and intubation in the delivery room were found to be significant predictors of surfactant therapy. This investigation adds to the current knowledge of biomarkers in preterm neonates with RDS, but further research is required to assess the predictive value of gastric fluid metabolomics in this field.

## 1. Introduction

Respiratory distress syndrome (RDS) is the most common respiratory disease in premature infants and is attributed to anatomical and functional immaturity of the neonatal lungs. The severity of RDS is inversely correlated with the degree of prematurity, and diagnosis is based on clinical manifestations, blood gas analysis, and imaging. Classically, neonates with RDS are treated with oxygen and mechanical ventilatory support (invasive or non-invasive), but exogenous surfactant administration is the cornerstone of treatment [1]. Despite all of the progress in perinatal medicine and neonatology, RDS is still frequently observed in very preterm infants [2] and is associated with severe complications including intraventricular hemorrhage, air-leak syndromes, bronchopulmonary dysplasia [1], and mortality [3].

The accurate prediction and prompt diagnosis of RDS is of paramount importance, but its development and evolution after birth is difficult to anticipate. Despite numerous studies performed during the last decades, no clinical or biological factor has been found to predict RDS early and accurately. Additionally, the classical clinical description of RDS has changed over the years, as its management has significantly evolved with the use of antenatal corticosteroids and the early initiation of nasal continuous positive pressure (CPAP) in the delivery room [4]. It should also be noted that when common clinical indicators of RDS development are used, disease progression has likely already occurred. At this point, the failure of supporting treatments (most commonly CPAP failure) is imminent and is associated with significant morbidity and mortality [5].

During the last years, oxygen requirement after birth has been proposed to guide surfactant replacement in preterm infants. According to the latest (2023) European Consensus Guidelines on the management of RDS, infants with RDS should be administered rescue surfactant early in the course of the disease—more specifically, those infants requiring a fraction of inspired oxygen (FiO_2_) > 0.30 while on a CPAP pressure of at least 6 cm H_2_O [6]. Lung ultrasound has also recently been proposed as a means of early identification of infants with RDS to help guide exogenous surfactant therapy [7].

Several laboratory techniques have also been used to evaluate surfactant activity in the amniotic or gastric fluid. These techniques are either quantitative (lecithin/sphingomyelin ratio, phosphatidyl/glycerol levels, and lamellar body counts) or qualitative (stable microbubble and surfactant adsorption tests) and are able to provide more personalized management of neonates with RDS [8,9].

In this context, we hypothesized that gastric fluid could potentially be an excellent biological specimen for the detection of candidate biomarkers related to lung maturation and to help guide the management of RDS. The use of advanced bio-analytical approaches, such as metabolomics, may play a key role in achieving this goal [10]. Metabolomic analysis of the amniotic fluid has been used for both the prediction of preterm delivery and bronchopulmonary dysplasia [11]. Recently, gastric fluid metabolites were found to predict survival in severe prematurity [12].

The purpose of the present study was to investigate whether metabolomic analysis of gastric fluid obtained early after birth in infants born before 32 weeks’ gestation could identify differences in intermediate metabolites between infants who require rescue surfactant therapy, and those who do not require replacement therapy and to assess if any of these metabolites could serve as an indicator of the need for surfactant therapy in very preterm infants with RDS.

## 2. Materials and Methods

### 2.1. Study Design and Population

This sub-study is part of a prospective, single-center, investigation on the role of metabolomics in the prediction of neonatal outcomes in very preterm infants (born at <32 weeks’ gestation), conducted from 1 March 2017 to 31 December 2020. Results describing the role of gastric fluid and urine metabolomic analysis obtained shortly after birth for the prediction of survival in very preterm infants have recently been published [12].

Herein, we analyzed data of the gastric fluid metabolomic analysis focusing on the need for surfactant replacement therapy for RDS. As previously described [12], we excluded neonates who were outborn; had known congenital infections, anomalies, or inborn errors of metabolisms; received chest compressions; and infants with missing gastric samples or those without parental consent. In the present study, infants who were administered surfactant prior to the sampling of the gastric fluid, either in the delivery room or very early after admission to the NICU, were also not included in the analysis.

We recorded variables related to neonatal demographical characteristics (gestational age, birth weight, sex and being small for gestational age), pregnancy (maternal age, multiple gestation, chorioamnionitis, hypertension/pregnancy-induced hypertension, antenatal corticosteroids), delivery mode, status after birth (Apgar scores at 1 and 5 min), intubation (in the delivery room (DR) and during the first 3 days of life), and receipt of exogenous surfactant in the NICU setting for the management of RDS (mode of administration and total doses). Neonates with blood culture-confirmed early-onset sepsis were recorded as well.

Cases were defined as neonates who were treated with exogenous surfactant for RDS, whereas the control group was made up of infants with mild or no RDS who did not receive surfactant. In our NICU, in line with the 2016 and 2019 updated European Consensus guidelines [13,14], infants with clinical signs of RDS (including increased oxygen requirements (FiO_2_ > 0.30) while on CPAP of at least 6 cm H_2_O) and compatible radiographic findings were administered rescue surfactant (poractant alpha) using either less invasive surfactant administration techniques or following endotracheal intubation and invasive mechanical ventilation. All infants were started on caffeine citrate from the first day of life.

### 2.2. Sampling

Gastric fluid samples were collected from the enrolled neonates with a thin gastric tube during the first hour of their life. Samples were stored at −80 °C until the metabolomic analysis.

### 2.3. Outcomes of Study

The main outcomes of this sub-study were: (1) to identify differences in the gastric metabolites in very preterm infants with RDS who received surfactant replacement compared to a control group of infants with mild or no RDS who did not receive surfactant; and (2) to evaluate the possible role of gastric fluids metabolites, alone or in combination with clinical variables, as indicators of the need for surfactant replacement therapy in preterm infants with RDS.

### 2.4. Analytical Methodology

Sample preparation and GC-MS analysis were applied, as previously described [12]. Briefly, the following steps were taken:

#### 2.4.1. Sample Preparation

All samples were thawed at room temperature before analysis. Initially, 50 μL of gastric fluid sample was mixed with 10 μL of myristic acid-d_7_ (internal standard, IS, Sigma-Aldrich, Merck Darmstadt, Germany) and 50 μL of ice-cold MeOH (−20 °C) (CHEM-LAB NV, Zedelgem, Belgium). The mixture was vortexed for 2 min and then centrifuged at 10,000 rpm for 15 min. Subsequently, 70 μL of the clear supernatant was transferred into a clear Eppendorf tube and evaporated under vacuum. Twenty-five microliters of MeOX 2% pyridine (Sigma-Aldrich (Merck Darmstadt, Germany) was added, followed by vortexing for 2 min and heating for 90 min at 30 °C. Then, 50 μL of MSTFA and 1% TMCS (Sigma-Aldrich, Merck Darmstadt, Germany) were added, and the sample was again heated for an additional 30 min at 37 °C. Finally, 10 μL of pentadecane (injection standard at 100 mg/L) was introduced, and the sample was subjected to GC-MS analysis.

A quality control (QC) sample was also prepared by mixing equal volumes of all analyzed real gastric samples (pooled sample). QC samples were analyzed at the beginning of the analytical batch and every ten real gastric fluid samples to ensure analytical performance.

#### 2.4.2. GC-MS Analysis

GC-MS analysis was performed in an Agilent 7890A GC-MS system (Agilent Technologies, Santa Clara, CA, USA), equipped with a CTC autosampler and a PTV injector. Gas chromatography was performed on a 30 m HP-5 ms UI (Agilent J&W) capillary column (film thickness of 0.25 mm; I.D. of 0.25 µm), while back-flush elution was carried out in a 1.5 m deactivated column with a film thickness of 0.18 mm. The initial column temperature was set at 60 °C for 1 min and then increased to 300 °C at a 10 °C/min rate. The temperature was maintained at 300 °C for 6 min. Total run time was 30 min, followed by a 12 min back-flush run and a solvent delay at 6 min. Helium (99.999%) was used as the carrier gas at a flow rate of 3 mL/min, and injection volume was set at 1 μL. Splitless mode injection was performed at the PTV injector, where the temperature was increased from 270 °C to 350 °C. MS was operated in electron impact ionization mode (EI; 70 eV). Ion source and transfer line temperatures were set at 230 °C and 250 °C, respectively. All mass spectra were acquired in full scan mode between 50 and 600 amu.

### 2.5. Statistical Analysis

The Kolmogorov–Smirnov test was used to assess the normality assumption. Continuous variables were described using the mean and standard deviation (SD) or median and interquartile range (IQR) according to the normality assumption. Frequencies (percentage) were used to describe categorical variables. The independent samples *t*-test and the Mann–Whitney U test were used to compare continuous variables between the two groups. The Pearson X^2^ test was used for the association between dichotomous variables. Crude and adjusted odds ratios (ORs) and 95% confidence intervals (CIs) for predicting the receipt of surfactant were calculated from univariate and multivariate logistic regression, respectively, followed by ROC/AUC calculation. An alpha-level <0.2 was selected as a cut-off for variable removal in the automated model selection, and backward elimination was preferred. All the statistical tests were two-sided, and the level of significance was set at a = 0.05. Data analysis was carried out using IBM SPSS Statistics, version 29.0 (IBM Corp., Armonk, NY, USA).

Gas Chromatography–Mass Spectrometry (GC-MS) data obtained from all analyzed samples were processed using MSD CHEMSTATION (G1701EA E.02.00.493) software. The Free Online Assignment Validator and Integrator (GAVIN) script for MATLAB (MathWorks, Natick, MA, USA) was employed for peak integration, complementing AMDIS for peak deconvolution and identification. Metabolite identification was based on the Agilent Fiehn library and NIST17 MS library (mainlib library), with a minimum match factor of 50%.

## 3. Results

### 3.1. Characteristics of Study Population

Seventy-three neonates were analyzed, comprising 43 cases and 30 controls. The perinatal–neonatal characteristics of the studied neonates are shown in Table 1. Compared to controls, cases had significantly lower gestational age and birth weight, as well as Apgar scores at 1 and 5 min, respectively. Moreover, cases were significantly more often intubated after delivery and received invasive mechanical ventilation in the NICU during the first three days after birth compared to infants in the control group (Table 1). In cases, an average of 1.8 (0.8) surfactant doses were instilled intratracheally, through an endotracheal tube (*n* = 19, 44.2%) or a catheter (*n* = 17, 39.5%), whereas in seven infants (16.3%), both approaches were used. No infant requiring intubation was administered prophylactic surfactant in the DR. One of the neonates treated with surfactant developed early-onset sepsis.

### 3.2. Differences in Gastric Fluid Samples

A detailed description of gastric fluid metabolites in neonates, including the differences between cases and controls, is presented in Supplementary Table S1. Statistically significant differences were observed in specific metabolites, namely L-proline, L-glycine, N-acetyl-L-serine, and L-threonine, all of which were increased in cases compared to controls (Table 2). Boxplots of the relevant metabolites are shown in Supplementary Figure S1. However, despite the observed differences, the AUC values obtained (Table 2) indicate that none of the metabolites alone could accurately predict the need for surfactant replacement.

### 3.3. Predictors of the Need for Surfactant Replacement Therapy

#### 3.3.1. Univariate Analysis

Gestational age, Apgar scores at 1 and 5 min, and intubation in the DR were significant predictors of the need for surfactant treatment in univariate analysis. Both gestational age and Apgar scores were considered negative predictors, associated with decreased probability for surfactant need for every 1 unit increase in their size [gestational age, (OR 0.70, 95% CI 0.54–0.91, *p* = 0.009), Apgar score at 1 min, (OR 0.70, 95% CI 0.511–0.94, *p* = 0.002) and Apgar score at 5 min, (OR 0.45, 95% CI 0.22–0.91, *p* = 0.026)] (Supplementary Table S2). In contrast, intubation in the DR, L-glycine, and acetyl-L-serine were associated with an 8.59-fold (OR 8.59, 95% CI 2.26–32.62, *p* = 0.002), 1.147-fold (OR 1.147, 95% CI 1.01–1.30, *p* = 0.029), and 1.143-fold (OR 1.143, 95% CI 1.01–1.29, *p* = 0.027) increase in the probability of receiving surfactant therapy, respectively.

#### 3.3.2. Multivariable Analysis

**Figure 1:**
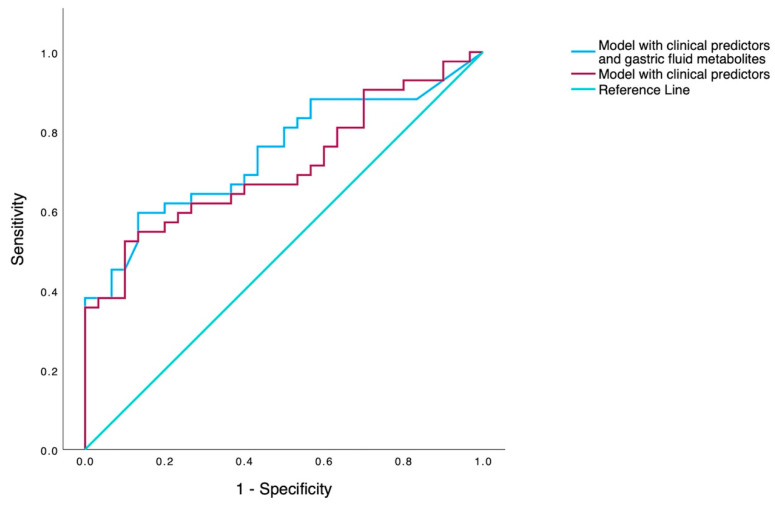
ROC curves from clinical predictors only (red line) and the combined model with gastric fluid metabolites and clinical variables.

Two multivariable models were constructed: a model including only significant clinical variables from univariate analysis, and a model with the combination of the above clinical variables and gastric fluid metabolites. In the first model, intubation in the DR remained the most potent predictor of the receipt of surfactant (OR 8.12, *p* = 0.002, 95% CI 2.14–31.75), demonstrating an AUC value of 0.69 (95% CI 0.57–0.81). When gastric fluid metabolites were incorporated into the multivariable model, the most accurate prediction for the receipt of surfactant therapy was accomplished with the inclusion of six variables: GA, Apgar scores at 1 and 5 min, intubation in the DR, acetyl-L-serine, and L-glycine. Among them, intubation in the DR and acetyl-L-serine were found to be the most significant, posing an 8.12-fold and 1.13-fold increased risk for surfactant receipt (Table 3), and the model demonstrated an AUC value of 0.76 (95% CI 0.64–0.86) (Figure 1).

## 4. Discussion

In this prospective study, we evaluated whether untargeted metabolomic analysis (GC-MS) of the gastric fluid in preterm neonates, obtained within the first hour of life, could predict the need for exogenous surfactant administration for the treatment of RDS. Although significant differences were observed between the two groups in gastric fluid metabolites (L-proline, L-glycine, L-threonine, and acetyl-L-serine), none could solely predict the need for surfactant replacement with high accuracy. Multivariate analysis, including significant clinical variables, demonstrated an AUC value of 0.69 (95% CI 0.57–0.81). The integration of metabolomic (L-glycine and acetyl-L-serine) and clinical data (gestational age, Apgar scores at 1 and 5 min, and intubation in the DR) in the multivariate analysis slightly improved accuracy (AUC 0.76, 95% CI 0.64–0.86) for the prediction of surfactant replacement therapy, but acetyl-L-serine and intubation in the delivery room were the only significant predictors of surfactant therapy.

### 4.1. Biochemical Role and Origin of Identified Metabolites

Very few studies have applied metabolomics in preterm infants with RDS to investigate their metabolic profile and possibly identify metabolic alterations/pathways associated with the pathophysiology of RDS. In these studies, bronchoalveolar lavage fluid (BALF) [15,16] and urine were used as specimens [17]. In the present study, gastric fluid was the biofluid of interest, in which four amino acids were found to be significantly higher in infants treated with surfactant compared to controls.

Nevertheless, the source and role of the above metabolites are difficult to interpret. They may represent biomarkers produced in the gastrointestinal tract of newborn preterm infants. On the other hand, the gastric fluid obtained that early after birth may still contain some quantity of amniotic fluid. Fetuses swallow large amounts of amniotic fluid every day. After birth, this is gradually emptied from the stomach due to gastric secretion and feeding [18]. Therefore, they could be metabolic products derived from the amniotic fluid, as glycine, proline, threonine, and serine have all been measured in human amniotic fluid [19,20]. L-glycine is produced from L-serine, a nonessential amino acid with a wide range of cellular functions, including the biosynthesis of proteins, sphingolipids, phospholipids (including phosphatidylserine), and folate [21]. Notably, L-glycine is involved in collagen production [22], being, thus, crucial for the structural integrity of various organs, including the lungs. Threonine is another essential amino acid related to glycine and serine [23]. It is also used in the biosynthesis of proteins, playing a critical role in the maintenance of intestinal mucosal integrity and barrier function [24]. In a recent study on metabolomics, Metwaly et al. reported significantly reduced serine, glycine, and threonine levels in the serum of critically ill adult patients with acute respiratory distress syndrome compared to ventilated controls, suggesting that the serine–glycine metabolic pathway may have dominant involvement in disease pathophysiology. To explain these metabolic derangements, the authors constructed the so-called “folate hypothesis” which, in brief, suggests that dysfunctional folate metabolism leads to low serine (the precursor to several amino acids) and disrupts mitochondrial redox homeostasis, causing oxidative stress injury. Moreover, low folate hampers cellular regeneration through impaired synthesis of pyrimidines and purines [25]. Proline is a non-essential amino acid with key roles in protein structure and function and in the maintenance of cellular redox homeostasis. It is either obtained through food or can be produced de novo within cells from protein structures, with collagen being a notable source [26].

L-serine needs special attention as it is involved in the synthesis of phosphatidylserine, one of the phospholipids present in pulmonary surfactant [27]. In general, deaminated amino acids, including glycine, serine, and threonine, following conversion to pyruvate, can serve as substrates for the Krebs cycle. The release of mitochondrial citrate into the cytoplasm ultimately leads, after several steps, to surfactant lipid and phospholipid synthesis [28]. Therefore, we speculate that the increased acetyl-L-serine found in cases may indicate altered surfactant metabolism due to lung immaturity. Nevertheless, it should be highlighted that we identified acetyl-L-serine, an acetylated derivative of the specific amino acid. Interestingly, this metabolite could be of microbial origin. For instance, it may be synthesized by *Escherichia coli* (*E. coli*), a common microorganism involved in intrauterine infection and preterm delivery, especially at very early gestational ages [29], such as in our study population. As previously documented, in a two-step process, serine acetyltransferase of *E. coli* catalyzes the O-acetylation of serine to O-acetyl-L-serine, in which sulfur is next assimilated, leading to the synthesis of cysteine [30]. In our case, however, we were unable to differentiate whether it was the N- or O-acetyl-L-serine. Moreover, there was no difference in the incidence of maternal chorioamnionitis or early-onset sepsis (both caused by bacterial infection) between the study groups to further support this speculation. Had we conducted microbiological studies of the obtained gastric fluid, we could have more confidently explained the association of gut microorganisms with the identified acetyl-L-serine.

In any case, none of the above gastric fluid metabolites could accurately serve as sole predictors for the need for surfactant treatment in infants with RDS. In another recent study, in which a similar approach was applied for the management of preterm infants with RDS, lamellar body count measured in the gastric aspirates was found to have moderate reliability in detecting CPAP failure (AUC 0.703) as well. Notably, the reliability of lamellar body count in predicting CPAP failure was even smaller (AUC 0.314) in the subgroup of neonates born at ≤32 weeks’ gestation [31].

### 4.2. Clinical Parameters to Prognosticate Surfactant Replacement Therapy

It is well recognized that the incidence and severity of RDS are inversely related to lung immaturity and, therefore, to gestational age and birth weight. In a study involving extremely preterm infants, surfactant was administered in 99.4% and 61.4% of cases born at 22 and 28 weeks’ gestation, respectively [32]. However, the decision to administer surfactant cannot be based on gestational age alone [33]. Regarding “intubation in the DR”, although we found this to be the most significant clinical indicator of surfactant use, its predictive value alone was only moderate (AUC 0.69). This may be explained by the fact that there are several reasons for endotracheal intubation of preterm infants immediately after birth, including resuscitation and the provision of invasive mechanical ventilation [2], as well as surfactant administration [6]. Interestingly, in an artificial intelligence model developed to predict RDS in very low birth weight infants, tracheal intubation at the initial resuscitation ranked last with respect to importance among the various maternal and perinatal–neonatal features evaluated [34]. Still, surfactant is currently recommended in intubated preterm infants, whether intubation was deemed necessary as part of initial stabilization or at any time to promote early extubation [6,33]. Notably, none of the study infants who required intubation immediately after birth were administered prophylactic surfactant in the DR. Moreover, to avoid the effect of this variable, we conducted a separate analysis after excluding infants intubated in the DR. No significant differences in gastric fluid metabolites were found in this separate analysis, so no further analysis was performed. This result was possibly due to the reduction in the sample size.

### 4.3. Integration of Metabolomic and Clinical Data

Unlike previously conducted relevant investigations in preterm infants, we explored the role of the significant metabolites along with other significant clinical parameters as predictors for the need for surfactant. Studies have shown that the integration of metabolomic and clinical data improves the accuracy of statistical models in predicting patient outcomes [35,36]. As concluded in a recent systematic review and network meta-analysis of 53 studies, in which clinical decision thresholds for surfactant administration in preterm infants were evaluated, further research into optimizing surfactant treatment for RDS should be performed, with a focus on practical surrogates of disease severity, including infants’ gestational age, co-morbidities and predisposing factors, modality and level of respiratory support, as well as FiO_2_ [37].

As a matter of fact, the combination of metabolomics (L-glycine and acetyl-L-serine) and clinical data (gestational age, Apgar scores at 1 and 5 min, and intubation in the DR) improved accuracy (AUC 0.76) in predicting the need for surfactant replacement. Nevertheless, the performance of our model is suboptimal compared to the FiO_2_-0.3 threshold (AUC 0.83) [5], lung radiograph (AUC 0.80), or ultrasound (AUC 0.95) [9].

### 4.4. Advantages and Limitations

To the best of our knowledge, this is the first study to evaluate metabolomic-based data for the identification of intermediate metabolites in the gastric fluid, which could possibly be used as predictors of the nee-d for exogenous surfactant in very preterm infants with RDS. Previous investigations (using mass spectrometry) only measured specific surfactant compounds (e.g., lecithin, sphingomyelin, phosphatidylglycerol, and surfactant protein A) in gastric aspirates as indicators of lung maturation [8]. However, there are disadvantages in our study as well. The fact that this investigation was limited to one center and therefore yielded a small sample size may have precluded the identification of other gastric fluid biomarkers with higher predictive accuracy. For this reason, we were unable to evaluate the effect of perinatal–neonatal parameters, such as intubation in the delivery room. Therefore, our results should be confirmed in the context of larger studies. Additionally, the application of other analytical platforms, such as lipidomics, might have allowed for the identification of metabolites associated with surfactant production and synthesis, such as lipids. Lastly, we acknowledge that despite the development of a good predictive model incorporating clinical parameters and metabolites, it offers limited advantages compared to simpler clinical indicators currently used in the management of preterm infants with RDS. In any case, it is our belief that this study will further contribute to future research surrounding the early identification of preterm infants who require surfactant replacement.

## 5. Conclusions

In conclusion, metabolomic analysis of gastric fluid aspirates obtained immediately after birth highlighted acetyl-L-serine as a possible biomarker, but metabolite profiling alone was not sufficient to identify preterm neonates with RDS requiring exogenous surfactant. The integration of metabolomic and clinical data improved the accuracy in predicting the need for surfactant replacement therapy. In any case, further research is required to explore the role of gastric fluid metabolomics in the diagnosis and management, not only of RDS, but also of other neonatal medical conditions and outcomes. With the future development of diagnostic kits that are easy to use in the NICU setting, metabolomics hold promise for the significant advancement in the optimal and individualized management of the sick neonate.

## Figures and Tables

**Table 1:** Perinatal and neonatal characteristics of cases and controls.

	Cases (*n* = 43)	Controls (*n* = 30)	*p*-Value *
Gestational age (weeks)	28 (4.0)	30 (2.0)	0.01
Birth weight (g)	1120 (670)	1240 (338)	0.036
Sex (male)	18 (41.9)	11 (36.7)	0.655
SGA	3 (7)	3 (10)	0.644
Multiple gestation	13 (30.2)	11 (36.7)	0.565
Maternal age (years)	32 (10.0)	35 (9.0)	0.243
Prenatal steroids (any)	41 (95.3)	29 (96.7)	0.780
Maternal MgSO_4_ administration	15 (34.9)	7 (23.3)	0.206
Chorioamnionitis (clinical or histological)	22 (51.2)	19 (63.3)	0.302
Mode of delivery-CS	37 (88.1)	27 (90)	0.80
Apgar score 1 min (median, IQR) Apgar score 5 min (median, IQR)	7.0 (4.5) 8.0 (1.0)	7.0 (1.0) 8.5 (1.0)	0.038 0.024
Hypertension/pregnancy-induced hypertension	5 (11.9)	2 (6.9)	0.487
Intubation in the DR	21 (48.8)	3 (10)	<0.001
IMV during the first 3 DOL	31 (72.1)	4 (13.3)	<0.001
Confirmed EOS	1 (2.3)	0	0.400

Quantitative variables are shown as median (IQR), and qualitative variables are shown as numbers (% percentage). * Results from Mann–Whitney *t*-test for continuous variables and X^2^-test for categorical variables. CS; cesarian section, DOL; day of life, DR; delivery room, EOS; early-onset sepsis, IMV; invasive mechanical ventilation, SGA; small for gestational age.

**Table 2:** Descriptive analysis and AUC values of the gastric fluid metabolites with significant differences between cases (*n* = 43) and controls (*n* = 30).

Metabolite	Group	Mean	SD	SE	Q 0.25	Q 0.50	Q 0.75	*p*-Value *	AUC (95% CI)
L-proline	Cases Controls	2.58 1.74	2.19 2.52	0.33 0.46	1.17 0.45	2.21 1.12	3.28 2.03	0.018	0.66 (0.53–0.79)
L-glycine	Cases Controls	6.05 3.39	5.35 3.73	0.81 0.68	2.67 0.63	4.50 2.59	8.80 5.07	0.036	0.64 (0.51–0.77)
Acetyl-L-serine	Cases Controls	6.34 3.52	5.62 3.80	0.85 0.69	2.88 0.68	4.65 2.73	8.97 5.31	0.036	0.65 (0.52–0.78)
L-threonine	Cases Controls	2.21 1.34	2.16 1.36	0.33 0.24	1.01 0.67	1.42 1.07	2.38 1.44	0.038	0.64 (0.51–0.76)

AUC: area under the curve, CI: confidence interval, SD: standard deviation, SE: standard error, Q 0.25, Q 0.50, Q 0.75: 25%, 50%, 75% percentiles of observations. * Results from Mann–Whitney *t*-test.

**Table 3:** Results from univariate and multivariate logistic regression, including perinatal–neonatal variables and gastric fluid metabolites for predicting the need for surfactant replacement therapy.

Variable	Univariate			Multivariate *		
	OR	*p*-Value	95% CI	OR	*p*-Value	95% CI
Gestational age (weeks)	0.70	0.009	0.54–0.91			
Apgar score at 1 min 5 min	0.70 0.45	0.022 0.026	0.51–0.94 0.22–0.91			
Intubation in the DR	8.59	0.002	2.26–32.62	8.12	0.003	2.07–31.84
L-glycine	1.15	0.029	1.01–1.30			
Acetyl-L-serine	1.14	0.027	1.01–1.29	1.13	0.045	1.01–1.27
L-proline	1.20	0.146	0.93–1.54			
L-Threonine	1.36	0.076	0.96–1.94			

OR: Odds ratio, CI: confidence interval, DR: delivery room, * Results from the final step of backward elimination from multivariate logistic regression.

## Data Availability

Data available on request due to restrictions such as privacy.

## Supplementary Materials

The following supporting information can be downloaded at: https://www.mdpi.com/article/10.3390/metabo14040196/s1. Table S1: Descriptive analysis of gastric fluid metabolites: differences between cases and controls and results of univariate logistic regression. Table S2: Univariate analysis for perinatal–neonatal predictors of the need for surfactant replacement. Figure S1: Boxplot of the significant metabolites in cases and controls.
