# Supplementary material for: Gastric Fluid Metabolomics Predicting the Need for Surfactant Replacement Therapy in Very Preterm Infants Results of a Case–Control Study
Source: Metabolites. 2024 Mar 30;14(4):196. doi: 10.3390/metabo14040196 (PMC11051721; doi:10.3390/metabo14040196)
Supplement: Supplementary file 1 [file metabolites-14-00196-s001.zip › Supp file 1. Supp Table S1.docx]

**Supp. Table S1.** Descriptive analysis of gastric fluid metabolites: differences between cases and controls and results of univariate logistic regression.

| **Metabolite** | **Cases**  **(mean, SD)** | **Controls**  **(mean, SD)** | **p-value*** | **Univariate OR** | **p-value**** | **95%CI** |
| --- | --- | --- | --- | --- | --- | --- |
| L-valine | 2.81 (3.86) | 2.13 (3.41) | 0.24 | 1.06 | 0.438 | 0.91 – 1.22 |
| 4-Methylcatechol | 0.09 (0.175) | 0.16 (0.365) | 0.68 | 0.39 | 0.355 | 0.05 – 2.83 |
| L-proline | 2.58 (2.18) | 1.74 (2.52) | **0.018** | 1.20 | 0.146 | 0.93 – 1.54 |
| 1-Methylhydantoin | 2.19 (1.58) | 1.79 (2.50) | 0.087 | 1.11 | 0.41 | 0.86 – 1.44 |
| Norleucine | 1.24(1.91) | 0.87 (1.58) | 0.25 | 1.14 | 0.383 | 0.84 – 1.54 |
| Glycerol | 4.79 (5.06) | 4.76 (4.68) | 0.76 | 1.00 | 0.976 | 0.90 – 1.10 |
| L-threonine | 2.21 (2.16) | 1.34 (1.35) | **0.038** | 1.37 | 0.076 | 0.97 – 1.94 |
| L-glycine | 6.05 (5.35) | 3.39 (3.72) | **0.036** | 1.15 | **0.029** | 1.01 – 1.30 |
| Acetyl-L-serine | 6.34 (5.62) | 3.52 (3.80) | **0.036** | 1.14 | **0.027** | 1.01 – 1.29 |
| Glyceric acid | 0.60 (0.45) | 0.50 (0.41) | 0.41 | 1.69 | 0.348 | 0.56 – 5.08 |
| Fumaric acid | 0.11 (0.08) | 0.09 (0.07) | 0.10 | 57.64 | 0.212 | 0.09 –33.55 |
| Quinaldic acid, 1,4-dihydro-6-nitro-4-oxo-, ethyl ester | 0.43 (0.42) | 0.27 (0.31) | 0.10 | 3.27 | 0.101 | 0.79 – 13.47 |
| Methylmalonic acid | 1.82 (2.11) | 1.48 (0.70) | 0.88 | 1.19 | 0.447 | 0.75 – 1.88 |
| L-serine | 0.01 (0.018) | 0.01 (0.016) | 0.65 | 154.74 | 0.726 | - |
| 5-oxo-L-proline | 3.88 (3.47) | 2.79 (2.40) | 0.26 | 1.14 | 0.149 | 0.96 – 1.35 |
| L-glutamic acid | 2.02 (1.56) | 1.55 (1.52) | 0.085 | 1.26 | 0.198 | 0.88 – 1.79 |
| L-threonic acid | 0.39 (0.26) | 0.31 (0.22) | 0.21 | 3.41 | 0.210 | 0.50 – 23.16 |
| Succinic acid | 0.18 (0.13) | 0.13 (0.10) | 0.27 | 19.87 | 0.137 | 0.38 – 1025.79 |
| 2,3,4-Trihydroxybutyric acid | 0.36 (0.23) | 0.32 (0.24) | 0.36 | 1.89 | 0.529 | 0.26 – 13.77 |
| Octanoic acid | 0.37 (0.36) | 0.30 (0.39) | 0.15 | 1.68 | 0.45 | 0.44 – 6.42 |
| D-xylose | 0.57 (0.78) | 0.57 (0.74) | 0.99 | 0.99 | 0.984 | 0.57 – 1.84 |
| 3,5-Di-tert-butyl-4-hydroxybenzoic acid | 0.32 (0.48) | 0.31 (0.43) | 0.99 | 1.05 | 0.927 | 0.38 – 2.90 |
| D-fructose | 0.63 (0.84) | 0.61 (0.76) | 0.95 | 1.02 | 0.941 | 0.57 - 1.83 |
| L-arabitol | 0.52 (0.29) | 0.48 (0.34) | 0.43 | 1.49 | 0.60 | 0.33 – 6.75 |
| D-galactose | 0.28 (0.25) | 0.26 (0.14) | 0.49 | 1.86 | 0.623 | 0.16 – 22.42 |
| 2,3-dimethylsuccinic acid | 1.32 (0.61) | 1.08 (0.59) | 0.16 | 1.95 | 0.113 | 0.86 – 4.43 |
| D-arabinose | 1.24 (1.07) | 1.35 (1.93) | 0.85 | 0.95 | 0.748 | 0.69 – 1.30 |
| 1-Deoxyglucose | 1.26 (1.08) | 1.352 (1.86) | 0.81 | 0.96 | 0.788 | 0.69 – 1.32 |
| 2-Phenoxathiinamine-10,10-dioxide | 0.56 (0.71) | 0.40 (0.23) | 0.44 | 2.09 | 0.295 | 0.53 – 8.25 |
| 17a-Aza-D-homo-5a-androstan-17-one | 1.77 (1.21) | 1.52 (1.13) | 0.36 | 1.20 | 0.389 | 0.80 – 1.80 |
| D-mannose | 5.61 (3.67) | 4.94 (3.53) | 0.36 | 1.05 | 0.434 | 0.92 – 1.21 |
| Myo-inositol | 6.37 (2.29) | 6.40 (2.18) | 0.96 | 0.99 | 0.951 | 0.81 – 1.23 |

OR; Odds ratio, CI; Confidence Interval

* p-value from Mann-Whitney t-test, ** p-value from univariate logistic regression
