# Supplementary material for: Gastric Fluid Metabolomics Predicting the Need for Surfactant Replacement Therapy in Very Preterm Infants Results of a Case–Control Study
Source: Metabolites. 2024 Mar 30;14(4):196. doi: 10.3390/metabo14040196 (PMC11051721; doi:10.3390/metabo14040196)
Supplement: Supplementary file 1 [file metabolites-14-00196-s001.zip › Supp file 2. Supp Table S2.docx]

**Supp. Table S2.** Univariate analysis for perinatal-neonatal predictors of the need for surfactant replacement.

| **Variable** | **OR** | **p-value** | **95%CI** |
| --- | --- | --- | --- |
| Gestational age (weeks) | 0.70 | 0.009 | 0.54-0.91 |
| Birth weight (g) | 0.99 | 0.06 | 0.997-1.001 |
| Sex (male) | 1.24 | 0.65 | 0.47-3.24 |
| Multiple gestation | 0.75 | 0.56 | 0.28-2.01 |
| SGA | 0.68 | 0.64 | 0.12-3.60 |
| Maternal age | 0.96 | 0.21 | 0.90-1.02 |
| Prenatal steroids (any) | 0.71 | 0.78 | 0.06-8.17 |
| ­Maternal MgSO_4_ administration | 0.50 | 0.21 | 0.17 – 1.47 |
| Chorioamnionitis (clinical or histological) | 0.60 | 0.30 | 0.23-1.57 |
| Mode of delivery-CS | 0.82 | 0.80 | 0.18 – 3.74 |
| Apgar 1min | 0.70 | 0.022 | 0.51-0.94 |
| Apgar 5min | 0.45 | 0.026 | 0.22-0.91 |
| Hypertension/pregnancy-induced hypertension | 1.82 | 0.49 | 0.32-10.12 |
| Intubation in the DR | 8.59 | 0.002 | 2.26-32.62 |

CI; Confidence Interval, CS; cesarian section, DOL; day of life, DR; delivery room, EOS; early-onset sepsis, IMV; invasive mechanical ventilation, OR; Odds ratio, SGA; small for gestational age
