# Supplementary figures and images for: Gastric Fluid Metabolomics Predicting the Need for Surfactant Replacement Therapy in Very Preterm Infants Results of a Case–Control Study
Source: Metabolites. 2024 Mar 30;14(4):196. doi: 10.3390/metabo14040196 (PMC11051721; doi:10.3390/metabo14040196)

**Supp. Fig S1.** Boxplot of the significant metabolites in cases and controls.


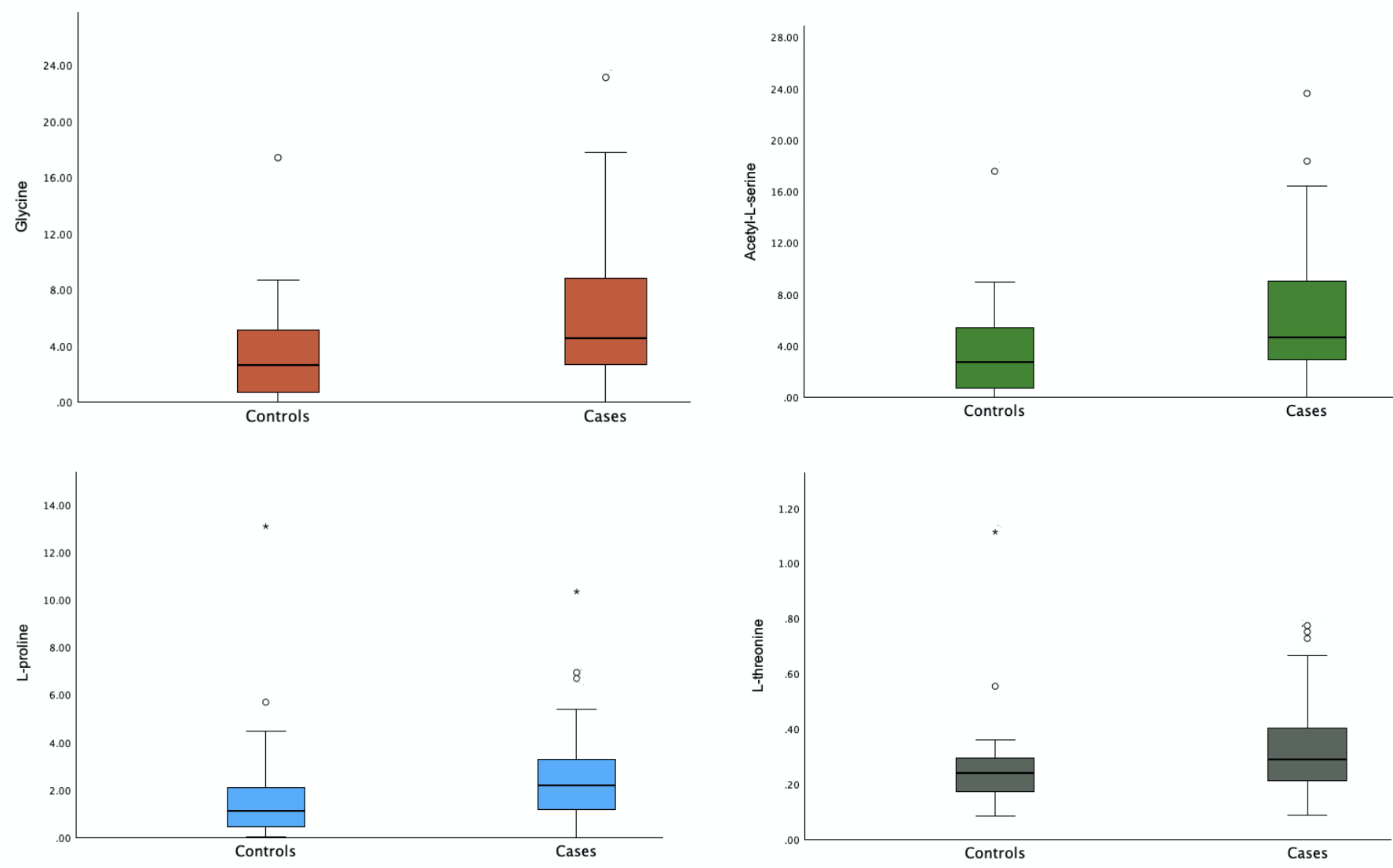

Supplement: Supplementary file 1 [file metabolites-14-00196-s001.zip › Supp. Fig S1.docx]
